# Prescribing Patterns for Upper Respiratory Tract Infections: A Prescription-Review of Primary Care Practice in Quetta, Pakistan and the Implications

**DOI:** 10.3389/fpubh.2021.787933

**Published:** 2021-11-19

**Authors:** Hania Hashmi, Nazeer Ahmad Sasoli, Abdul Sadiq, Abdul Raziq, Fakhra Batool, Shanaz Raza, Qaiser Iqbal, Sajjad Haider, Syed Umer Jan, Muhammad Alam Mengal, Abdul Malik Tareen, Adnan Khalid, Fahad Saleem

**Affiliations:** ^1^Faculty of Pharmacy and Health Sciences, University of Baluchistan, Quetta, Pakistan; ^2^Department of Surgery, Bolan Medical Complex Hospital, Quetta, Pakistan; ^3^Department of Biochemistry, Jhalawan Medical College Khuzdar, Khuzdar, Pakistan; ^4^Department of Statistics, University of Baluchistan, Quetta, Pakistan; ^5^Department of Pharmacy, Sardar Bahadur Khan Women's University, Quetta, Pakistan; ^6^Center for Advanced Studies in Vaccinology and Biotechnology, University of Baluchistan, Quetta, Pakistan; ^7^Department of Microbiology, University of Baluchistan, Quetta, Pakistan; ^8^Department of Surgery, Combined Military Hospital, Quetta, Pakistan

**Keywords:** prescribing patterns, upper respiratory tract infections, prescription-review, Quetta city, Pakistan

## Abstract

**Background:** To identify and address the potential overuse of antibiotics, it is important to ascertain the prescribing practices of physicians. We, therefore, conducted this prescription analysis to document URTI-specific antibiotic prescription frequency in a public primary healthcare setting of Quetta city, Pakistan.

**Methods:** A retrospective record review was conducted of all prescriptions for URTIs in Combined Military Hospital, Quetta from 1 March to 31st May 2021. The Mann-Whitney U and Jonckheere–Terpstra test was used to evaluate the association between the tendencies of a different group of prescribers. *p*-value of <0.05 was of statistical significance.

**Results:** Over the 3 months, 50,705 prescriptions were screened and analyzed according to the established inclusion and exclusion criteria. A total of 4,126 (8.13%) URTI prescriptions met the inclusion criteria, of which 2,880 (69.80%) prescriptions contained antibiotics. Among all antibiotics, penicillins (Amoxicillin + Clavulanate) were the most prescribed antibiotic, constituting 1,323 (45.9%) of total antibiotics prescribed for all cases, followed by the Macrolide group 527 (18.2%). The Jonckheere–Terpstra test revealed a statistically significant association between the status of the prescriber and the diagnosis (*p* = 0.002). Furthermore, a moderate positive trend was reported with specialists being more competent in antibiotic prescribing based on their diagnosis, followed by postgraduates and house officers (τ = 0.322).

**Conclusion:** The prescribing patterns for the management of URTIs in the hospital were inconsistent with current guidelines. Strict adherence to guidelines must be ensured and antibiotic prescribing for URTIs should be discouraged.

## Introduction

Upper respiratory tract infections (URTIs) are acute infections involving the nose, paranasal sinuses, pharynx, larynx, trachea, and bronchi (1). Often mild and self-limiting in nature, URTIs occasionally lead life-threatening complications (2). Primarily caused by the Rhinovirus (3, 4), 0.5–10% of the reported cases are because of Group A Streptococci (1). Therefore, physicians must differentiate viral and bacterial pictures while establishing an effective therapeutic plan for patients with URTIs (5). However, defining and differentiating such patients is difficult because the clinical presentations connected with bacterial or viral-related URTIs commonly overlap (6), hence antibiotics are frequently prescribed to manage URTIs in primary care settings (7–11). Nevertheless, and other than certain exceptions, antibiotics are unnecessarily prescribed for URTIs (12). This frequent use of antibiotics adds a burden to healthcare systems that result in clinical failure and/or an increase in the development of antibiotic resistance (13).

The inappropriate and over-prescribing of antibiotics in ambulatory care is frequently reported in the literature (11, 14, 15). Rowe and Linder claimed that most antibiotic use in the US occurs in ambulatory care and 30–50% is inappropriately prescribed to the patients (16). Zhao et al. (17) in their nationwide study also reported inappropriate antibiotic prescribing in China where >50% of the antibiotic prescriptions were inappropriate at the tertiary-level hospitals. Another study revealed that ambulatory care physicians in the US wrote almost 12 million prescriptions for URTIs and acute bronchitis, of these 51% of adults with colds were prescribed an antibiotic, 52% in non-specific URTIs, and 66% in acute bronchitis (18). The hysterical and indiscriminate antibiotic use in ambulatory care has increased the risk of resistance development that is further augmented by their low cost and easy accessibility (18–21). As a result, common infections are now becoming more difficult to treat with standard antibiotics, forcing a shift to newer generations of antibiotics, which are more specific and targeted, but more expensive, and with a higher level of side effects (22–25).

Quality use of antibiotics is getting worse in the Asian region. Antibiotics consumption between the years 2000–2015 increased from 3.2 to 6.5 billion DDDs (103%) in India, from 2.3 to 4.2 billion DDDs (79%) in China, and 0.8 to 1.3 billion DDDs (65%) in Pakistan (26). Routine microbiologic cultures and sensitivity testing is often not performed, antibiotic therapy is empirical, and the few available antibiotics are overused or misused. This increases the emergence and spread of resistance and, therefore sub-optimal clinical outcomes (27, 28). Shifting our concerns to antibiotic use in Pakistan, the country is facing a huge crisis when quality use of antibiotics is discussed in the literature. Augmented by the data supplied by Quintiles-IMS, the Center for Disease Dynamics, Economics and Policy (CDDEP) reported an increasing sales trend and suggested a rise in consumption of almost every antibiotic in Pakistan (29). However, the data covered sales of antibiotics registered for human use only and there is no information about antibiotic use in animals or the agricultural sector. Very recently Bilal et al. (30) reported high resistance to commonly used antibiotics and identified gaps in surveillance and breaches in methodological data. The information was available from only two provinces of the country and no data was available from the other provinces of Pakistan.

Pakistan despite having an essential drug list is facing issues of lacking standard guidelines for the treatment of infectious diseases (31). In Pakistan, data suggest that in tertiary care hospitals junior doctors tend to follow the prescriptions of senior or specialist doctors, yet for them, standard treatment guidelines are non-existent to guide clinical decisions (31, 32). To develop a national antibiotic policy or infection control policy, data on antibiotic prescribing patterns in various infections reporting to different tertiary care public and private hospitals are need-fully required.

Correlating irrational use of antibiotics in URTI, large variations in antibiotics prescribed for URTIs exist that are difficult to explain (33). Within this context, the patterns of antibiotic prescription show that it has a huge impact on treatment outcomes (34). However, prescribing an antibiotic is a complex task that requires diagnostic skills, knowledge of antibiotics, understanding of the principles of clinical pharmacology, communication skills, and the ability to make decisions based on judgments of potential benefit and risks, having considered available evidence and specific factors relating to the patient being treated. Prescribing an antibiotic for URTIs is fundamental where the knowledge of physicians with proper training on antibiotics is highly needed. Additionally, factors including patient' age, religious beliefs, comorbidities, adherence to treatment guidelines, and financial status influence the prescribing patterns of antibiotics (35). Therefore, the guide to good prescribing highlights the selection and evaluation of appropriate drug therapy and consider medication cost when prescribing (36). Consequently, prescribing practices play an important role in deciding the success of the therapy and therapeutic outcomes. Consequently, we conducted this prescription analysis to document URTI-specific antibiotic prescription frequency in a public primary healthcare setting of Quetta city, the provincial capital of Baluchistan province.

## Methods

### Study Design and Prescription Selection Criteria

A retrospective prescription analysis was conducted whereby all prescriptions from 1st March 2021 to 31st May 2021 were screened and retrieved for further investigation. Prescriptions with mentioned diagnosis of “URTI,” “tonsillitis,” “pharyngitis,” “rhinitis,” “common cold,” “sore throat,” “cough,” or “otitis media” were included in the study (1). Incomplete prescriptions, missing diagnoses, or prescriptions with more than one infection were excluded from the study as we wanted to minimize the uncertainty of the diagnosis hence the purpose of prescribed antibiotics.

### Identification of URTI Diagnosis

URTIs were defined based on the most agreed criteria (1). All prescriptions were in hard copies, retrieved manually from the Outpatient Departments (OPDs) of the hospital. Because of the unavailability of symptomatology or laboratory results, the validity of diagnosis was not viable and hence we selected the prescriptions solely on the written diagnosis on the prescription. The prescriptions were screened manually by the first author, who is a qualified and practicing pharmacist and has considerable experience and competence in this regard.

### Classification and Appropriate Prescribing of Antibiotics

A comprehensive guideline for the management of Respiratory Tract Infections is provided by the Medical Microbiology & Infectious Diseases Society of Pakistan (37). However, specific instruction on the management of URTIs is not available in Pakistan. Subsequently, we evaluated prescribing practices based on the recommendations of the National Institute for Health and Care Excellence (38). The classification of antibiotics used in this study was adapted from 2019 WHO AWaRe Classification Database of Antibiotics for evaluation and monitoring of use (39).

### Study Settings and Sampling

The research was conducted at the Out-Patient Department of Combined Military Hospital (CMH), Quetta. Combined Military Hospital is a tertiary care teaching hospital situated in Quetta Cantonment and is operated by Pakistan Armed Forces. After British colonization, it was established as British Military Hospital (BMH) in 1854, which was converted to the Indian Army Medical Corps (IAMC) in 1927. After partition in 1947, it was handed over to Pakistan Army and was named CMH. Combined Military Hospital is one of the biggest hospitals of the city and in the access of the public. All departments are well established here with facilities and modern machinery.

### Statistical Analysis

The data was coded and entered into Statistical Package for the Social Sciences (SPSS), version 21 for further analysis. The Kolmogorov-Smirnov test was used for testing the normality of the sample distribution. Both descriptive and inferential statistics were used for data elaboration. Frequencies and percentages were used to summarize the data. The Mann-Whitney test was used to associate dichotomous variables. The Jonckheere–Terpstra test was used to evaluate the trend of association between the tendencies of a different group of prescribers. Where significant associations were reported, the effect size was calculated by using the Kendall tau correlation coefficient. *p*-value of < 0.05 was of statistical significance.

### Ethical Approval

The ethics committee of the Faculty of Pharmacy & Health Sciences, University of Baluchistan, Quetta approved the study. Permission for data collection was also taken from the Commandant CMH, Quetta. Being a record review, consent for publication was not required.

## Results

Over the 3 months, 50,705 prescriptions were screened and analyzed according to the established inclusion and exclusion criteria. A total of 4,126 (8.13%) URTI prescriptions met the inclusion criteria, of which 2,880 (69.80%) prescriptions contained antibiotics. Nearly 40% of the prescriptions were diagnosed as non-specific URTI followed by cough (694, 16.8%) and rhinitis (491, 11.9%). Thirty percent of prescriptions were from the pediatrics unit and the majority (1,664, 78.3%) of the prescription with URTIs were prescribed by postgraduates (Table 1).

**Table 1:** Study characteristics by age, prescriber status, and diagnosis.

**Characteristics**	**Number of prescriptions with confirmed URTIs**	**Number of prescriptions with antibiotics, *N* (%)**
**Patients' age (years)**		
> 10	1,145	951 (83.0)
19-Oct	856	553 (64.6)
20–29	512	385 (75.1)
30–39	687	412 (59.9)
40–49	475	315 (66.3)
50,59	388	224 (57.7)
> 60	63	40 (63.4)
**Prescriber status**		
House officers	654	514 (78.5)
Postgraduates	2,125	1,664 (78.3)
Specialists	1,347	702 (52.1)
**Prescribing OPD**		
Family	774	18.8
Pediatrics	1,268	30.7
ENT	402	9.7
General medicine I	727	17.6
General medicine II	558	13.5
General medicine III	397	9.6
**Diagnosis**		
Non-specific URTI	1,648	39.9
Cough	694	16.8
Sore throat	406	9.8
Rhinitis	491	11.9
Pharyngitis	472	11.4
Tonsillitis	157	3.8
Otitis media	79	1.9
Sinusitis	149	3.6
Nasopharyngitis	30	0.7

*OPD, outpatient department, ENT, ears, nose, and throat*.

Table 2 presents the frequency of antibiotics prescribed to patients. Among all antibiotics, penicillins (Amoxicillin + Clavulanate) were the most prescribed antibiotic, constituting 1,323 (45.9%) of total antibiotics prescribed for all cases, followed by the Macrolide group 527 (18.2%). In terms of prescribing, most of the patients were prescribed Amoxicillin + Clavulanate after consultations with postgraduates, compared to specialists and house officers respectively.

**Table 2:** Choice of antibiotics prescribed for upper respiratory tract infections.

**Antibiotic class**	**Anatomical therapeutic chemical code**	**Name of antibiotic**	**Prescribed for URTI**	**Prescribed by house officers**	**Prescribed by postgraduates**	**Prescribed by specialists**
			***N*** **(%)**	***N*** **(%)**	***N*** **(%)**	***N*** **(%)**
Penicillins	J01CA	Amoxicillin+clavulanate	1,323 (45.9)	57 (4.3)	1,026 (77.5)	240 (18.1)
		Amoxicillin	260 (9.0)	2 (0.7)	251 (96.5)	7 (2.6)
Macrolides	J01FA	Clarithromycin	311 (10.8)	56 (18.0)	157 (50.4)	98 (31.5)
		Azithromycin	198 (6.8)	14 (7.0)	144 (72.7)	40 (20.2)
		Erythromycin	18 (0.6)	8 (44.4)	9 (50.0)	1 (5.5)
Cephalosporins	J01DB/C	Cefixime	459 (15.9)	11 (2.3)	417 (90.8)	31 (6.7)
		Cefaclor	33 (1.1)	0	17 (51.5)	16 (48.4)
Tetracyclines	J01AA	Doxycycline	13 (0.4)	0	8 (61.5)	5 (38.4)
Sulfonamide	J01EE	Co-trimoxazole	20 (0.6)	1 (5.0)	8 (40.0)	11 (55.0)
Quinolones	J01MA	Levofloxacin	185 (6.4)	22 (11.8)	90 (48.6)	73 (39.4)
		Moxifloxacin	40 (1.3)	0	29 (72.5)	11 (27.5)
		Ciprofloxacin	32 (1.1)	8 (25.0)	11 (34.3)	12 (37.5)

Among all URTI cases, Amoxicillin + Clavulanate was the most favored antibiotic in more than 50% of cases, except for sinusitis and nasopharyngitis. Levofloxacin was preferred as the treatment of choice in sinusitis (12.1%) while Cefixime was prescribed for Nasopharyngitis (20%). The antibiotic prescription against specific diagnoses is described in Table 3.

**Table 3:** Antibiotic prescription against specific diagnosis.

**Diagnosis**	**Cases prescribed with antibiotic (*N*)**	**Types of prescribed antibiotics**					
		**Amoxicillin + clavulanate**	**Amoxicillin**	**Clarithromycin**	**Azithromycin**	**Erythromycin**	**Cefixime**
Non-specific URTI	1,648	579 (35.1)	160 (9.7)	77 (4.7)	108 (6.6)	4 (0.2)	246 (14.9)
Cough	693	156 (22.5)	39 (5.6)	124 (17.9)	26 (3.8)	8 (1.2)	32 (4.6)
Sore throat	406	207 (51.0)	16 (3.9)	18 (4.4)	6 (1.5)	0	19 (4.7)
Rhinitis	491	80 (16.3)	22 (4.5)	12 (2.4)	16 (3.3)	0	59 (12.0)
Pharyngitis	472	166 (35.2)	13 (2.8)	58 (12.3)	25 (5.3)	5 (1.1)	51 (10.8)
Tonsillitis	157	70 (44.6)	2 (1.3)	11 (7.0)	7 (4.5)	1 (0.6)	19 (12.1)
Otitis media	79	28 (35.4)	0	5 (6.3)	0	0	14 (17.7)
Sinusitis	149	33 (2.1)	4 (2.7)	6 (4.0)	6 (4.0)	0	13 (8.7)
Nasopharyngitis	31	4 (13.3)	4 (13.3)	0	4 (13.3)	0	6 (20.0)
Non-specific URTI	1,648	23 (1.4)	3 (0.2)	3 (0.2)	69 (4.2)	5 (0.3)	7 (0.4)
Cough	693	1 (0.1)	2 (0.3)	6 (0.9)	45 (6.5)	17 (2.5)	8 (1.2)
Sore throat	406	0	2 (0.5)	0	12 (3.0)	0	0
Rhinitis	491	4 (0.8)	2 (0.4)	0	12 (2.4)	5 (1.0)	4 (0.8)
Pharyngitis	472	2 (0.4)	2 (0.4)	2 (0.4)	57 (5.7)	5 (1.1)	0
Tonsillitis	157	3 (1.9)	2 (1.3)	0	0	0	6 (3.8)
Otitis media	79	0	0	0	0	3 (3.8)	6 (7.6)
Sinusitis	149	0	0	9 (6.0)	18 (12.1)	5 (3.4)	0
Nasopharyngitis	31	0	0	0	2 (6.7)	0	0

The Jonckheere–Terpstra test revealed a statistically significant association between the status of the prescriber and the diagnosis (*p* = 0.002). Furthermore, a moderate positive trend was reported with specialists being more competent in antibiotic prescribing based on their diagnosis, followed by postgraduates and house officers (τ = 0.322). No significant association, however, was reported among other study variables.

## Discussion

Quality use of antimicrobials and an increased frequency of antimicrobial resistance (40) have emerged as a major health crisis. Antimicrobial resistance has spread to almost all countries and regions, including Pakistan, owing to the indiscriminate use of antibiotics and poor infection control practices. Several factors contribute to the development of AMR and among those irrational prescribing, free availability of antibiotics, and patient-related factors are commonly highlighted in the literature. Within this context, Sulis et al. (40), in their meta-analysis concluded that antibiotics are highly prescribed in primary care and there is a need for urgent action to improve prescription practices, starting from the integration of WHO treatment recommendations and the AWaRe classification into national guidelines. Therefore, the primary objective of the current study was to assess the prescribing practices of physicians while managing patients with URTIs in a primary healthcare setting of Pakistan. Although prescribing practices for URTIs are reported from other parts of Pakistan, the current study is the first piece of evidence reported from the province of Balochistan. Furthermore, our focus was strictly on prescribing practices for URTIs, and that is what we managed to achieve, makes this study different from others as they reported both upper and lower respiratory tract infections.

Our study highlighted frequent use of antibiotics (69.80%) for URTIs from Quetta city, Pakistan, and the published literature provides mixed results in this context. By and large, the prescription rate for URTI in the current study was higher than the rates observed from the Asian region. Antibiotics were prescribed to 51.6% of the patients in Bahrain (40) and 31.8% of patients in Malaysia (11). In Japan, antibiotics were prescribed to 60% of the patients diagnosed with URTIs (41). However, the antibiotic prescribing rate in URTIs in this study was lower than what is reported in other studies. John et al. (42) reported that almost 88% of prescriptions contained antimicrobials for the treatment and management of acute tonsillitis in the UAE. Also, a multi-center study in Pakistan reported that 88.9% of the prescriptions contained antibiotics for the treatment and management of URTIs (43). The differences in rates could be explained by different natures of the denominator used in these studies as well as the study setting, data collection period, and the difference in the types and availability of antibiotics. Additionally, patients' expectations or demands of an antibiotic during the consultation are also frequently reported in the literature as a major reason for inappropriate antibiotic prescribing.

As documented in the literature and guidelines, only a very limited number of patients with URTIs warrant antibiotic treatment (38, 44, 45). However, this study has found antibiotics were frequently prescribed for non-specific URTIs, cough, and rhinitis (Table 3). Within this context, The Centers for Disease Control and Prevention (CDC) provides clear criteria for physicians when diagnosing URTIs. The presence of tonsillar exudates, tender anterior cervical adenopathy, history of fever, and lack of cough is an indication that antibiotics are not required under such conditions (46). For that reason, appropriate clinical judgment is fundamental while ascertaining bacterial etiology before antibiotics are prescribed in URTIs. Parallel to this measure, Rezal et al. (11), suggested that developing and practicing local antibiotic guidelines and continuous medical education regarding antibiotic use also make a huge difference in rational antibiotic prescribing. Using such measures will help make the right medication choices and dosing. Eventually, a successful reduction in antibiotic prescription will result in a rapid drop in AMR. The Australian initiative toward the use of quinolones through its national pharmaceutical subsidy scheme is an excellent example whereby this policy has successfully preserved the utility of this class of antimicrobial drugs for the treatment of most infections (47). The efficiency of practicing guidelines for antibiotic prescribing is also evident in literature whereby by reducing macrolides prescription in Japan, a decline in resistance rate from 22 to <2% of group A streptococcal isolates was reported (48).

The trend of association reported specialists being more competent in antibiotic prescribing based on their diagnosis, followed by postgraduates and house officers. From the clinical perspective, the reported trend is comprehensible. As experience increases, healthcare professionals also expand their skills and knowledge while practicing safe patient care. Also, experienced healthcare professionals are often more prepared mentally and are equipped with proficiency in dealing with a medical crisis. In line with what is being discussed, Lewis et al. (49), in their qualitative study reported that among junior doctors, knowledge, and expertise played a key role in prescribing mistakes. Krishnakumar and Tsopra (50) also mentioned personal factors such as experience and knowledge while choosing a particular antibiotic in clinical conditions. We must remember that antibiotic prescribing is a complex, context-dependent, and dynamic process that entails the balancing of many tensions. Other than that, we also believe that the variations in prescribing between the different prescribers in the current study settings are attributed to some other factors. Where biomedical factors provide key assistance while selecting an antibiotic, factors ranging from attitudes of the prescribers and patients to managerial constraints and policies can also influence the prescribing decision. Our claims are supported by the meta-ethnography published by Wojcik et al. (51) whereby the authors reported that antibiotic prescribing is an intricate phenomenon and comprehensive efforts are needed to promote the distribution of responsibility for antibiotic decisions. Consequently, it is high time that policymakers need to take steps to address these issues. Potential next steps should include continuous medical education for the house officers and implementation of stewardship programs focusing on strict compliance of guidelines implementation.

## Conclusion

The prescribing patterns for the management of URTIs in the hospital were inconsistent with current guidelines. Quality use of antibiotics can help prevent the emergence of AMR; consequently, a better understanding of appropriate antibiotic prescribing must be fostered among prescribers. Strict adherence to guidelines must be ensured and antibiotic prescribing for URTIs should be discouraged. We also urge the policymakers to introduce antimicrobial stewardship programs and guidelines in healthcare institutes that will help with planning future initiatives among the primary healthcare centers of Pakistan.

## Limitations

In a single-centered study, the generalizability of the findings is always an issue. Also, the diagnoses of URTIs were based on the written diagnosis on the prescription and we did not verify the accuracy of the clinical examination and diagnosis with the prescribers due to the reasons described earlier. We, therefore, recommend a comprehensive study involving multiple (public and private) healthcare institutes with a confirmed diagnoses of URTI in consultations with the prescribers.

## Data Availability Statement

The raw data supporting the conclusions of this article will be made available by the authors, without undue reservation.

## Ethics Statement

The Ethics Committee of the Faculty of Pharmacy and Health Sciences, University of Baluchistan, Quetta approved the study. Permission for data collection was also taken from the Commandant CMH, Quetta. Written informed consent was not required in this study in accordance with the national legislation and the institutional requirements.

## Author Contributions

HH, NS, AS, and AR conceptualized and designed the study. FB, SR, QI, and SH collected the data while SU, MM, and AT analyzed and interpreted the data. The study was supervised by AK and FS. All authors have met the criteria for authorship and had a role in preparing the manuscript. Also, all authors approved the final manuscript.

## Conflict of Interest

The authors declare that the research was conducted in the absence of any commercial or financial relationships that could be construed as a potential conflict of interest.

## Publisher's Note

All claims expressed in this article are solely those of the authors and do not necessarily represent those of their affiliated organizations, or those of the publisher, the editors and the reviewers. Any product that may be evaluated in this article, or claim that may be made by its manufacturer, is not guaranteed or endorsed by the publisher.
